# Enhanced Cassava Flour Quality to Improve the Cassava Bread by Attibutes Yeast Fermentation

**DOI:** 10.1002/fsn3.70581

**Published:** 2025-07-14

**Authors:** Peixu Du, Jinxiao Liang, Hao Zhang, Wanbi Tan, Guotao Liang, Onguala Paul, Quanfa Zhou, Liming Lin, Xingxing Dong, Zhenwen Zhang, Yi He

**Affiliations:** ^1^ National R&D Center for se‐Rich Agricultural Products Processing, Hubei Engineering Research Center for Deep Processing of Green se‐Rich Agricultural Products, School of Modern Industry for Selenium Science and Engineering Wuhan Polytechnic University Wuhan China; ^2^ Key Laboratory for Deep Processing of Major Grain and Oil, Ministry of Education, Hubei Key Laboratory for Processing and Transformation of Agricultural Products, School of Food Science and Engineering Wuhan Polytechnic University Wuhan China; ^3^ Tropical Crops Genetic Resources of Institute Chinese Academy of Tropical Agricultral Science/National R&D Center for Patato Processing Haikou China; ^4^ ACRO Biotech Co. Ltd. Zhuhai China; ^5^ Ministry of Agriculture, Pastoral and Fisheries of the Democratic Republic of the Congo Agricultural Demonstration Centre of Techniques Kinshasa Democratic Republic of the Congo

**Keywords:** cassava bread, cassava flour, fermentation, nutritional quality, physicochemical properties, volatile compounds

## Abstract

Cassava is rich in starch and serves as a food staple in tropical and subtropical regions worldwide. However, cassava contains hydrocyanic acid, which can be harmful to human health in high concentrations. Proper processing methods, such as soaking, fermentation, or cooking, are typically employed to reduce cyanide levels to safe limits (< 10 mg HCN/kg), where it may even exhibit antioxidant properties. Without adequate processing, excessive consumption of cassava with high cyanogenic content may pose health risks. Yeast fermentation improves the nutritional and functional properties of cassava flour, improves protein content, reduces hydrocyanic acid content, increases flavor compounds, and alters starch particle size, viscosity, and relative crystallinity. These changes contribute to better dough elasticity, increased nutrient bioavailability, and overall quality improvement in cassava bread. The results demonstrated that the nutrition of fermented cassava flour was enhanced, with the protein content increasing from 0.89 ± 0.08 g/100 g to 3.86 ± 0.12 g/100 g and the hydrocyanic acid content decreasing from 32.64 ± 0.67 mg/kg to 7.40 ± 0.22 mg/kg. Fermentation significantly altered the volatile profile of cassava flour, increasing both the diversity and concentration of flavor compounds. GC–MS analysis detected 17 volatile components in fermented cassava flour, compared to only 12 in unfermented flour (control). Key flavor‐active compounds—including 2‐methyl‐1‐butanol (malty), phenethyl alcohol (floral), and ethyl linoleate (fruity)—were notably enhanced, contributing to the improved aroma of fermented cassava flour. The findings indicated that yeast fermentation enhanced cassava flour's nutrition (protein) and flavor compounds and improved its processing characteristics. Fermentation also modified the physicochemical properties of cassava flour, including reduced particle size, lower paste viscosity, and a more ordered crystalline structure—changes that enhance its processing stability (e.g., resistance to retrogradation and improved dough handling). When incorporated into bread formulations, 20% fermented cassava flour yielded superior textural properties (e.g., reduced hardness, higher elasticity) and higher sensory scores (appearance, flavor, and overall acceptability) compared to cassava breads and very close to wheat bread.

## Graphical abstract

- Yeast fermentation enhances cassava flour's nutrition, increasing protein from 0.89 ± 0.08 to 3.86 ± 0.12 g/100 g, reducing hydrocyanic acid from 32.64 ± 0.67 to 7.40 ± 0.22 mg/kg, and boosting flavor compounds.
- It modifies physicochemical properties: smaller particle size, lower paste viscosity, more ordered crystal structure, and improved processing stability.
- Bread with 20% fermented cassava flour has better texture, higher sensory scores, and is close to wheat bread.

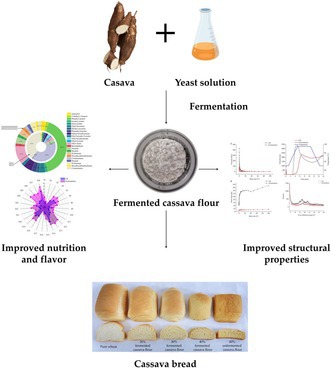

## 1 Introduction

Cassava is a tuberous edible crop mainly grown in Asia, Africa, Central and South America, and Oceania (Abass et al. 2018). It has many varieties, low requirements for planting conditions, and can grow in poor soil (El‐Sharkawy 2014). Cassava has the highest yield of carbohydrates per hectare of any crop and can be grown at a considerably lower cost (Chisenga et al. 2019), which is a staple food in many developing countries and regions, with a starch content of approximately 30% in fresh cassava root and approximately 70% in dried ones (Okpako et al. 2008). Cassava starch is a digestible starch comprising 20% amylose and 80% amylopectin but lacking nutrients (protein, amino acid, fat) and flavor. Meanwhile, it also contains a small amount of cyanogenic glycosides, which are hydrolyzed to form hydrocyanic acid, a toxic substance that induces Konzo disease when contents are greater than 10 mg/kg (Ahaotu et al. 2017). Konzo is mainly seen in children and women of childbearing age and is associated with a high intake of hydrocyanic acid from cassava (Banea et al. 2014). The traditional method of cassava food preparation is to remove the skin, dry it, cook it, or soak it in water to eliminate the cyanogenic glycosides. For example, in South America, Indonesia, and other Southeast Asian countries, cassava flour is used to make bread, cakes, and other foods after fermentation (Lu et al. 2020).

Yeast is a very favorable and safe fermentation fungus, which is rich in protein, amino acids, and other nutrients. It can also degrade starch and cellulose to produce lactic acid, citric acid, and other substances (Sampaolesi et al. 2019). On the other hand, microbial fermentation can reduce the hydrocyanic acid content and improve the nutrient content of cassava flour, thereby increasing the characteristic flavor of cassava flour (Sobowale and Oyewole 2008). Meitha et al. (2016) found that microbial fermentation affects the physicochemical properties of cassava flour and reduces the hydrocyanic acid content. However, systematic studies on yeast fermentation's effect on enhancing nutrients and flavor of cassava flour and related product development still need to be completed.

In this study, cassava flour was subjected to yeast fermentation and analyzed for changes in its nutrients, volatile compounds, and physicochemical properties. We have also developed cassava bread based on fermented flour, which tries to provide a theoretical basis for the development and utilization of nutritious, healthy, and safe cassava food by fermentation.

## 2 Materials and Methods

### 2.1 Experimental Materials

South China 9 (SC9) cassava fresh root was harvested from the National Cassava Germplasm Resource Nursery, Institute of Tropical Crop Variety Resources, Chinese Academy of Tropical Agriculture. Anqi highly active dry yeast powder (Saccharomyces yeast) (Yichang, China) was commercially purchased from the Jingdong Supermarket. Fresh cassava was peeled (inner and outer skin), washed, and ground into cassava homogenization using a potato grinder (YL8022, Good Luck Mechanical and Electrical Equipment Co., Hunan Province, China). Cassava homogenization (200 g), Anqi highly active dry yeast powder at 5% (v/v) inoculation amount, and 200 mL of sterile water taken from an ultrapure water machine (PGE‐Z‐10J, Pinguan Instrument & Equipment Co., Wuhan, China) were added together to a conical flask, stirred well, sealed with sealing film, and placed in a constant temperature culture shaker (HZ250LB, Yiheng Scientific Instruments Co., Shanghai, China) at 25°C and 220 rpm for fermentation. The fermented cassava homogenization (200 g wet weight) was collected after 24 h of fermentation and dried to constant weight in an oven (Blue Pard, Yiheng Scientific Instruments Co., Shanghai, China) at 50°C for 48 h. The dried material was then milled using a laboratory grinder and sieved through a 100‐mesh (150 μm) stainless steel sieve to obtain uniform fermented cassava flour. Meanwhile, cassava homogenates that were not subjected to inoculated fermentation were dried and powdered as CK.

### 2.2 Protein Content

According to the method described by Waterborg, the protein content was detected (Waterborg 2009). The protein assay was performed by adding 0.1 mL of 2 N NaOH to 0.1 mL sample/standard, followed by hydrolysis at 100°C for 10 min. After cooling to room temperature, 1 mL of freshly prepared complex‐forming reagent was added and incubated for 10 min. Subsequently, 0.1 mL Folin reagent was added with vortex mixing, and the reaction mixture was incubated at room temperature for 30–60 min (not exceeding 60 min). Absorbance was measured at 750 nm (for protein concentrations < 500 μg/mL) or 550 nm (100–2000 μg/mL). Protein concentrations were determined against a standard curve of absorbance versus known protein concentrations.

### 2.3 Amylose Content

According to the manufacturer's instructions, the amylose content was measured using the ‘Amylose Content Kit’ (Research & Development Biotechnology Co., Shanghai, China). Briefly, samples were homogenized and mixed with the provided extraction buffer, followed by centrifugation to collect the supernatant. The supernatant was then reacted with the kit's reagent at 100°C for 10 min, cooled to room temperature, and mixed with iodine solution. The absorbance was measured at 620 nm using a spectrophotometer, and the amylose content was calculated based on a standard curve prepared with known amylose concentrations.

### 2.4 Reducing Sugars Content

According to the Friedemann et al. method, the reducing sugar content was detected (Friedemann et al. 1962). The reduced sugar content was determined using the following formula:

(1) $$V=\frac{\left(V_{0}-V_{1}\right)\times C}{0.1}$$

where, ‘V’ is the volume of 0.1 mol/L potassium ferricyanide solution required to oxidize the reducing sugars in the sample solution (mL), ‘V_0_’ is the volume of 0.1 mol/L sodium thiosulfate solution used in titrating the blank solution (mL), ‘V_1_’ is the volume of 0.1 mol/L sodium thiosulfate solution used in titrating the sample solution (mL), ‘C’ is the actual concentration of sodium thiosulfate solution (mol/mL).

### 2.5 Soluble Sugars Content

According to the Clegg method, the soluble sugars content was detected (Clegg 1956). Briefly, finely ground samples (0.2 g) were extracted twice with hot 80% ethanol, and the combined supernatants were evaporated to remove ethanol. The residue was diluted to a concentration of ~100 μg glucose/mL. For analysis, 1 mL of the extract was mixed with 1 mL of water (test) or 100 μg glucose standard (internal control), followed by 10 mL of anthrone reagent (1 g/L in 76% H_2_SO_4_). After heating at 100°C for 12 min, absorbance was measured at 620 nm. Sugar content was calculated using the standard curve and dilution factor. The soluble sugar content was determined using the following formula:

(2) $$V=\frac{C\times V}{m}$$

where, ‘X’ is the mass fraction of soluble sugar (%), ‘C’ is the mass concentration of soluble sugar (mg/mL), ‘V’ is the volume of sample liquid (mL), and M is the mass of the sample (mg).

### 2.6 Crude Fat Content

According to the Smith and Tschinkel method, the crude fat content was detected (Smith and Tschinkel 2009). The crude fat content was determined using the following formula:

(3) $$F\left(\%\right)=\frac{\left(m_{2}-m_{1}\right)}{m}$$

where, ‘m’ is the mass of the sample (g), ‘m_1_’ is the mass of the extraction bottle (g), ‘m_2_’ is the mass of the extraction bottle and fat (g).

### 2.7 Hydrocyanic Acid Content

The differences in the hydrocyanic acid content were determined using an isonicotinic acid‐pyrazolone spectrophotometric assay, according to Qin's method (Qin et al. 2021).

### 2.8 Flavor Compounds

Differences in the volatile and flavor components between CK and fermented cassava flour were determined by gas chromatography (GC)‐mass spectrometry (Agilent 7890/5975C GC/MSD, Agilent Technologies, USA) combined with an electronic nose (cNose‐18, Baosheng Industrial Development Co., Shanghai, China).

The samples were weighed, sealed with cling film in a small beaker, heated in a water bath at 45°C for 30 min, and tested by manual headspace injection using an electronic nose. The test conditions were as follows: carrier gas, clean and dry air; sampling interval, 1 s; cleaning time, 100 s; zero adjustment time, 10 s; presampling time, 5 s; measurement time, 100 s; sensor chamber flow rate, 400 mL/min; measurement sample flow rate, 400 mL/min; and maximum 3.0 g.

The samples were placed in a solid‐phase microextraction vial and equilibrated on a solid‐phase microextraction bench in a water bath at 50°C for 30 min. After the volatiles had equilibrated with the upper air and the lower solution, the activated extraction head was inserted and pushed out to expose it to the headspace of the vial for extraction and sorption for 30 min. When the sample was equilibrated, the fiber tip was retracted, the sorbed extraction needle was quickly inserted into the GC inlet, and the fiber tip was pushed out and desorbed at 250°C for 5 min. Simultaneously, GC was initiated. The sample was analyzed, and data were collected. The operating parameters were as follows: column: DB‐5MS column (50 m × 0.25 mm × 0.25 μm), carrier gas: ultra‐pure helium, flow rate: 1.0 mL/min, injector temperature: 250°C, initial oven temperature: 40°C, programmed to 230°C at the rate of 5°C/min, injection volume: 1 μL in split mode at a split ratio of 30:1, electron ionization potential: 70 eV, ion source temperature: 250°C, and scan range: 30–500 amu.

### 2.9 Physicochemical Properties

1. Particle size: The particle sizes of cassava flour were determined using a laser particle size distribution instrument (BT‐9300, Baite Instruments Co., Dandong, China). Pure water was used to clean the measurement vessel several times automatically. After deducting the background, the analytical model was chosen as a spherical model. Cassava flour was added gradually in small amounts to several different parts of the vessel containing pure water, and the stirrer dispersed the samples until the refractive index was in a particular range to start the measurement of the samples. In the experiment, D_10_, D_50_, and D_90_ denote the particle sizes corresponding to the cumulative size distribution of the samples at 10%, 50%, and 90%, respectively. The results were processed using the BT‐9300 Laser Particle Sizer software.
2. Flow sheet properties: The flow sheet properties of cassava flour were measured using a rheometer (CP5000, Laimei Technology Co., Guangzhou, China). Viscosity was measured using a rapid viscometer (RVA‐Super4, Perten Instruments, Stockholm, Sweden). Samples were weighed (3.000 g, dry basis) in a sample box, added with 25 mL of water, held at 35°C for 3 min, heated to 95°C at a rate of 6°C/min, held for 5 min, and cooled to 50°C at a rate of 6°C/min. Viscosity was measured, and the peak curve was plotted.
3. Crystallinity: X‐ray spectra of CK and fermented cassava flour were obtained using an X‐ray diffractometer (BDX3200, Tsingtao Sky Bridge Instrument Co., Beijing, China). Crystallinity was determined before and after fermentation at a scan speed of 0.05°/min and a scan angle range of 4°–40° as a function of 0.02° (2θ) as follows:

(4) $$\mathrm{RC}\left(\%\right)=\frac{100\times A_{c}}{\left(A_{c}+A_{a}\right)}$$

where, ‘RC’ is the relative crystallinity, ‘A_c_’ is the crystalline region, ‘A_a_’ is the amorphous region.

### 2.10 Cassava Bread and It's Characteristics

1. Making cassava bread: Fermented cassava flour (20%; 30%; 40%) was added to wheat flour to make the bread following the procedure. A certain amount of wheat flour, cassava flour (20%; 30%; 40%), yeast (1%), sugar (5%), salt (1%), and water (65%) was weighed with a balance and put into a dough machine (LC‐XNJ10, Guangdong Lechon Electrical Appliance Co., Guangdong, China), and the dough was formed with a smooth surface without cracks after 7 min, and then put the dough into a waking box with a waking temperature of 35°C–37°C and a humidity of 80%–85% to waken up for 30 min, and the softened butter was added into the dough for 7 min to make the dough again. Sprinkle flour on a lightly floured surface to prevent sticking. Roll the dough into a circle, cover with a damp muslin cloth, and let rise for 5 min. Divide the dough into two equal portions (about 100 g) using a floured cutter, fold the portions in half to eliminate air bubbles, and let rise for 5 min. Then, pull the portions into long strips of uniform thickness, fold the long sides in half, fold the edges downwards, and cover with a damp muslin cloth. Fold the strip in half, face upwards, roll out the strip with a rolling pin to form a sheet of even thickness, press out any air bubbles with the palm of your hand, roll up the short side of the sheet, and place it in a mold (160 × 90 × 75 mm) with a little butter on the bottom. Place the mold in the oven for 2 h. Preheat the oven (NB‐WJH3202, Panasonic (China) Co., Beijing, China) 20 min in advance and bake the bread at 175°C on the top and 215°C on the bottom for 30 min, switching the sides and adjusting the direction at the 15th min to ensure even heating. After baking, take the bread from the oven and let it cool for 2 h at room temperature.
2. Specific volume of bread: After cooling, the bread was weighed, and its volume (cm^3^) was determined by the canola seed replacement method. Specific volume (cm^3^/g) was calculated using bread volume/bread weight.
3. Awakening height of bread: Dough Proofing Height Measurement: Place dough in a marked container, record initial height (H_0_), then measure at intervals (H_1_) until optimal rise or collapse. Calculate expansion (ΔH = H_1_ – H_0_).
4. Bread texture: Test parameters of the TPA mode of the mass spectrometer: 25 N induction element, P/100 probe, speed 1.0 mm/s before test, 3.0 mm/s during test, 3.0 mm/s after test, 50% compression, induction force (trigger force) 5 g (0.05 N), time between two compressions 3 s, three tests for each group of samples, and took the average value (Yu et al. 2019).
5. Sensory evaluation of bread: Based on the references with slight modifications, eight trained food professionals were selected to perform sensory evaluation of the finished bread on the following scores (Yu et al. 2019) (Table 1).

**TABLE 1:** Evaluation of sensory score on cassava bread.

Sensory index	Scoring standard	Scores
Shape	Complete shape, no collapse	20–30
	Asymmetry in shape	10–19
	Severe shape change	0–9
Color	Creamy bright color	10–20
	Golden bright color	5–9
	Dark gray without light color	0–4
Internal organization	Stomata are fine in size	20–30
	Uneven size of stomata	10–19
	Extremely uneven size of stomata	0–9
Flavors	Strong flavor	10–20
	Not a strong flavor	5–9
	No flavor or odor	0–4

### 2.11 Statistical Analysis

Each test is repeated three times. The data are presented as mean ± standard deviation (SD). Data were analyzed by analysis of variance (ANOVA), with a confidence interval of 95%, and tested for normality first. The data processing programs used are IBM SPSS Statistics 19 and Origin 2018.

## 3 Results and Discussion

### 3.1 Changes in Nutrients and Hydrocyanic Acid Contents

As shown in Table 2, the protein, soluble sugar, and crude fat contents of cassava flour increased significantly after fermentation (*p* < 0.01). Related studies have shown that yeast fermentation can increase the protein content of cassava flour. The elevated protein content could be attributed to extracellular enzymes secreted by yeast cells, coupled with nitrogen enrichment in the fermentation medium (Khurshida et al. 2025). While amylose content showed no significant change, reducing sugar and the hydrocyanic acid contents decreased significantly after fermentation (*p* < 0.01). The observed reduction in amylose leaching may be attributed to yeast‐mediated formation of amylose complexes, which could inhibit the fragmentation of swollen starch granules and enhance their structural stability (Tester and Morrison 1990). Fermentation can also reduce the hydrocyanic acid content of cassava (Damayanti et al. 2021). In our study, the hydrocyanic acid content after fermentation was 7.40 mg/kg, which was reduced to 23% of the initial value (32.64 mg/kg), which is lower than the World Health Organization safety limit of 10 mg/kg (Ampe et al. 1994). The significant reduction in hydrocyanic acid content after yeast fermentation is due to the secretion of cyanide hydrolase by yeast during fermentation, which interacts with formamide hydrolase to decompose cyanogenic glycosides into carbon dioxide, ammonia, and formic acid (Gupta et al. 2010).

**TABLE 2:** Changes of components for cassava flour.

Main components	CK	Fermentation
True protein (g/100 g)	0.89 ± 0.08	3.86 ± 0.12**
Amylose starch (g/100 g)	10.12 ± 0.11	10.08 ± 0.17
Reducing sugar (g/100 g)	0.45 ± 0.04	0.24 ± 0.03**
Soluble sugar (g/100 g)	0.07 ± 0.01	0.18 ± 0.03**
Crude fat (g/100 g)	0.28 ± 0.02	0.79 ± 0.01**
Hydrocyanic acid (mg/kg)	32.64 ± 0.67	7.40 ± 0.22**

*Note:* Data are expressed as mean ± standard deviation (SD) (*n* = 3). CK is for unfermented cassava flour, fermentation is for fermented cassava flour.

** Indicates significant differences at *p <* 0.01.

### 3.2 Analysis of Volatile Components and Flavor Composition

**FIGURE 1:**
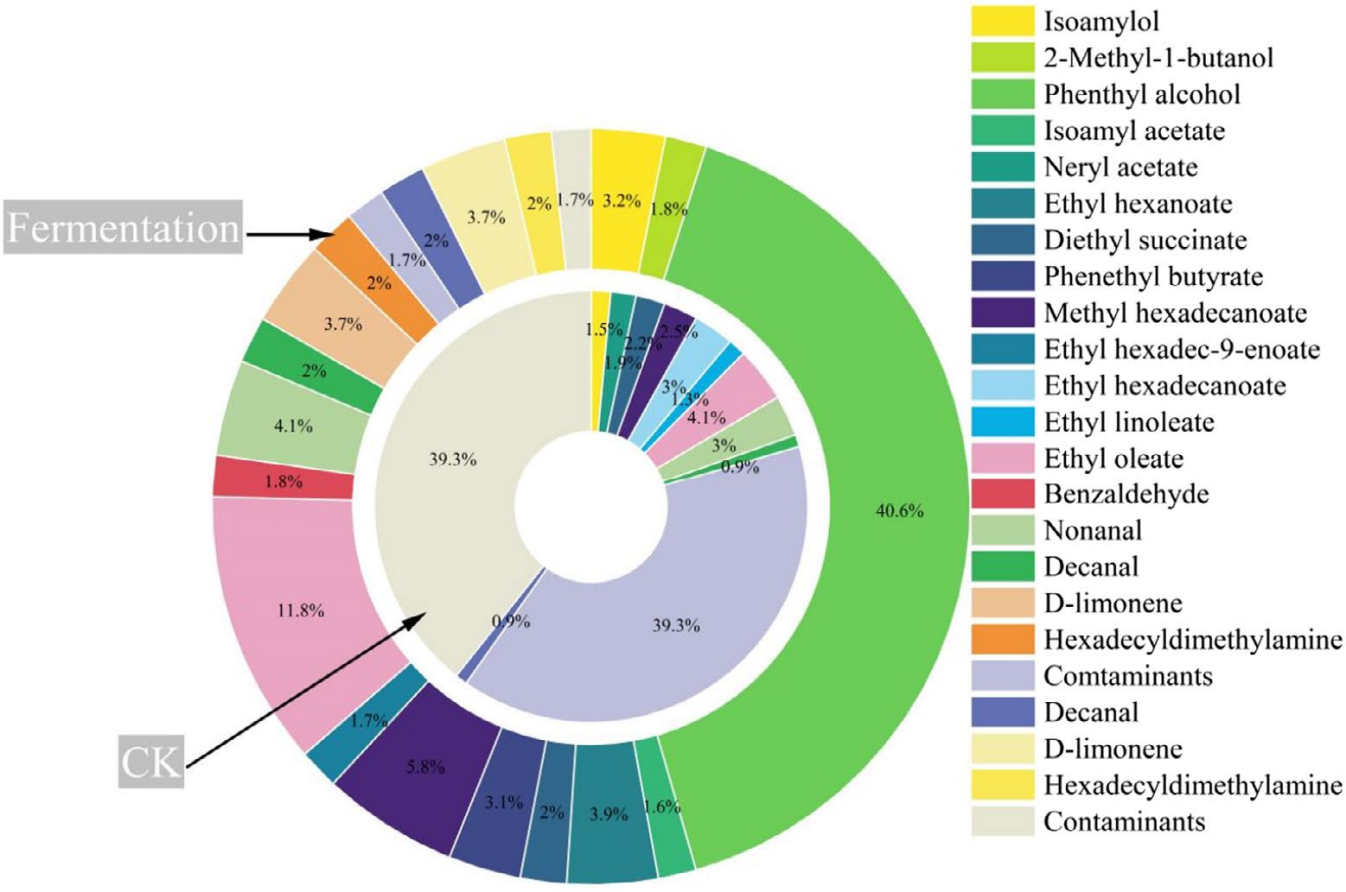
Comparison of volatile components for cassava flour.

**FIGURE 2:**
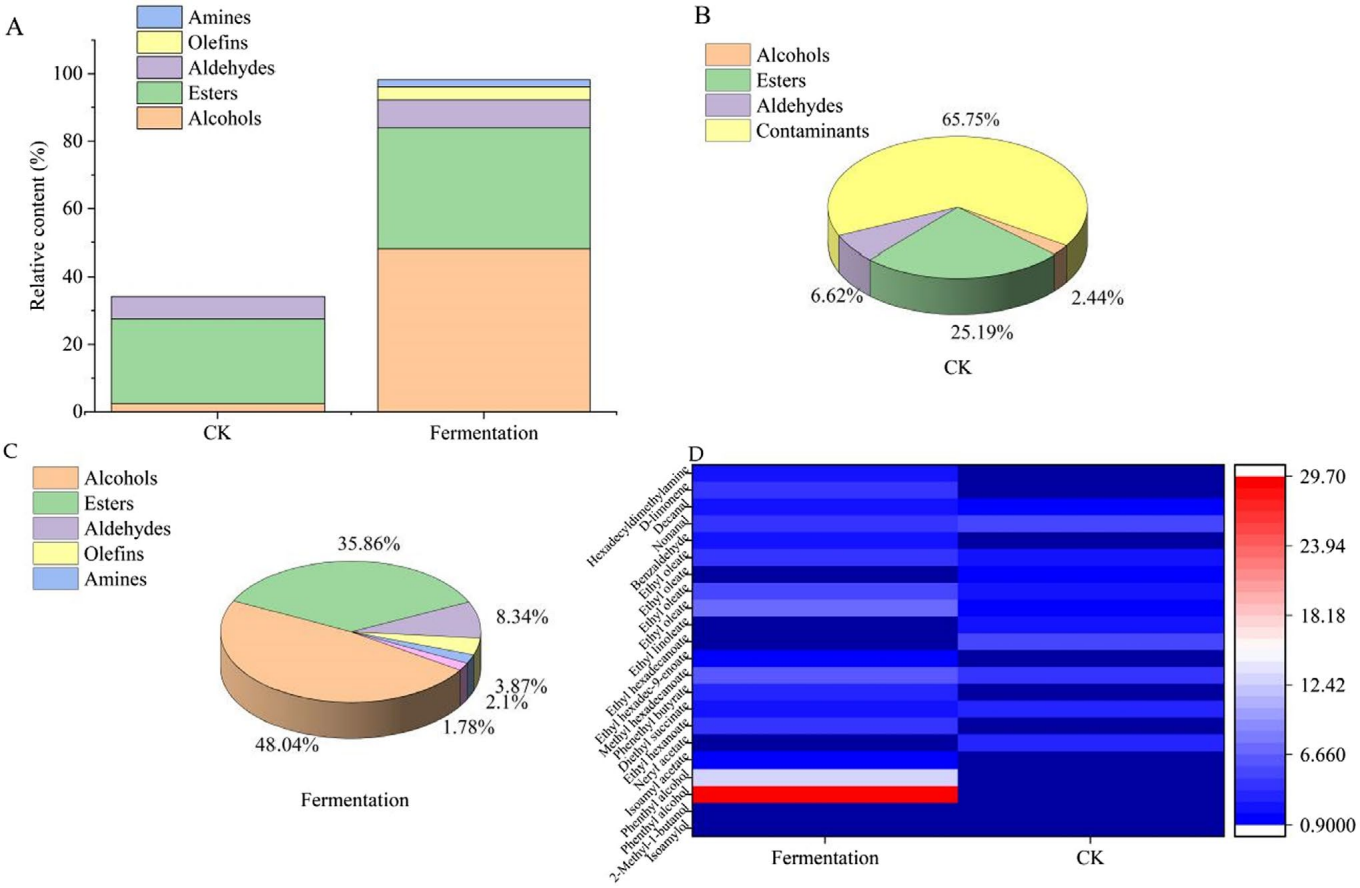
Comparison of volatile components for cassava flour. (A) Histogram of volatiles accumulation of CK and Fermented cassava flour, (B) Pie chart of volatiles of CK, (C) Pie chart of volatiles of Fermented cassava flour, (D) Heat map of CK and Fermented cassava flour.

The total relative content of volatile components detected in fermented cassava flour reached 98.22%, while CK was < 50% (Figure 1), and a total of 20 volatile compounds were detected in cassava flour, including alcohols, esters, aldehydes, olefin, and amine (Figure 2A). Through qualitative analysis, 17 volatile components were detected in fermented cassava flour, whereas only 12 were detected in CK; alcohols and esters showed significant changes after fermentation among the common components (Figure 2B,C). Among the main volatile compounds of fermented cassava flour, several alcohols were identified, such as 2‐methyl‐1‐butanol and phenethyl alcohol (Figures 1 and 2D). 2‐Methyl‐1‐butanol, produced through the leucine‐derived Ehrlich pathway (Chen 1978), contributes distinct malty aroma notes. With its low odor threshold, this compound becomes organoleptically significant even at trace concentrations in fermented cassava systems. Among these is ethyl linoleate, which gives cassava flour its strong pineapple fruit and floral aroma. However, few studies have examined the volatile compounds of fermented cassava flour. Most existing studies have focused on analyzing the volatile components present in cassava wine (Coelho et al. 2020). Thus, further investigations are desirable in this area of research.

**FIGURE 3:**
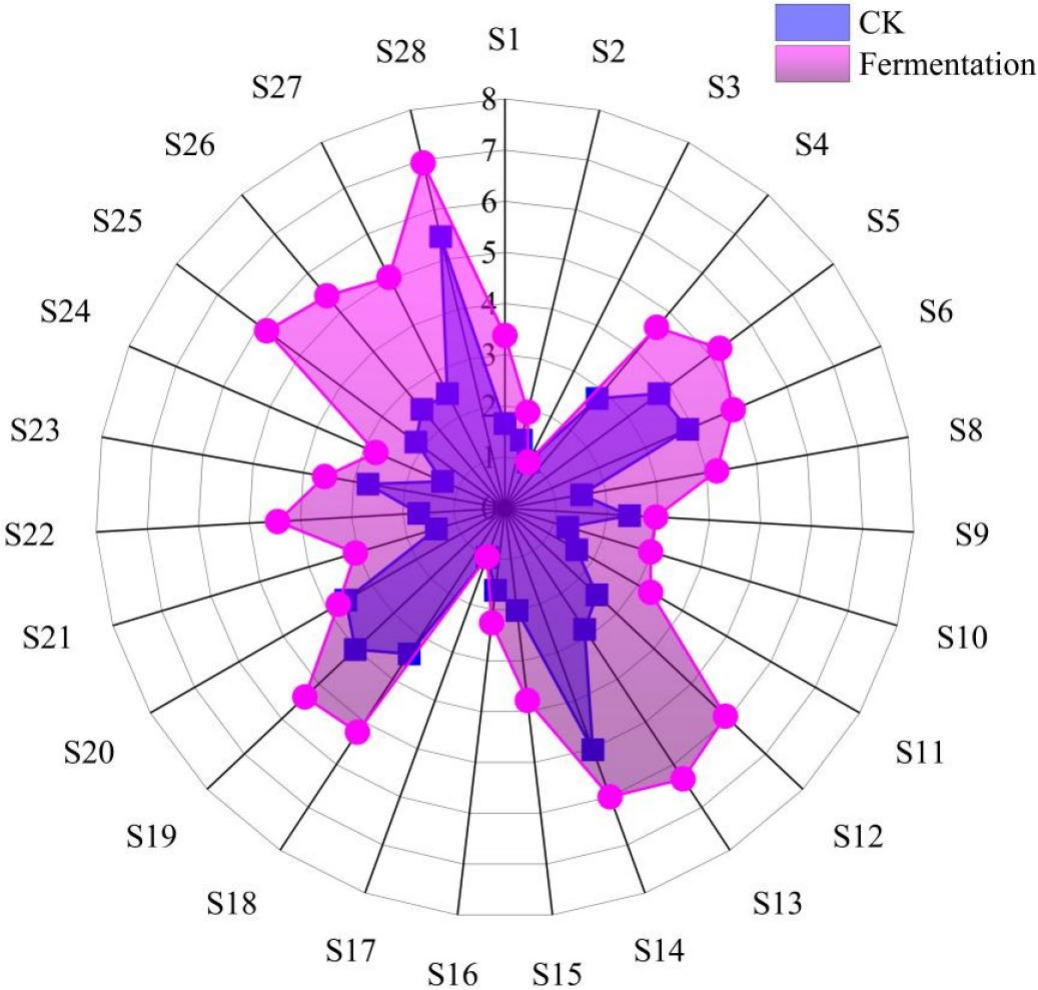
Comparison of flavor composition for cassava flour.

The overall response values increased to different degrees, except for the S3 and S17 sensor response values (Figure 3; Table S1), which decreased from those before fermentation. The S3 and S17 response substances were hydrogen and short‐chain alkanes, which were not produced under the fermentation conditions in this study. The rest of the sensors are basically esters, alcohols, sulphides, etc. The response intensities and response substances of each sensor of CK and fermented cassava flour were generally consistent with GC–MS and some previous findings (Coelho et al. 2020; Lu et al. 2020; Swiegers et al. 2005). Yeast fermentation converts most sugars in cassava flour into alcohols, esters, aldehydes, etc., which is mainly the effect of the production of alcohols, thus affecting the aroma of cassava flour (Kataoka et al. 2000). A study on cassava flour and cassava flour biscuits revealed volatile components in cassava biscuits (detected using an electronic nose) (Lu et al. 2020) that are similar to those identified in the present study, but the flavor components were not as rich as those found in the present study on fermented tapioca flour and lacked the improved alcohol and ester flavors. A study (Arroyo‐López et al. 2009) investigated the effects of yeast and bacteria on wine. They found that the moderating effect of yeast and bacteria on wine aroma and flavor increased the production of yeast fermentation and concluded that yeast fermentation caused the type, content, and aroma threshold of volatile aroma compounds to interact harmoniously with one another, which eventually led to the characteristic aroma of fermented cassava flour. In summary, the results of the combined GC–MS and electronic nose studies showed that yeast fermentation of cassava flour has a significant enhancing effect on its odor.

### 3.3 Characterization of Cassava Flour Processing

#### 3.3.1 Particle Size

From Table 3, it can be seen that fermentation affected the particle size distribution of cassava flour, and the D_10_, D_50_, and D_90_ of fermented cassava flour decreased by 20.1%, 16.8%, and 15.9%, which indicates that the fermentation of yeast significantly reduced the particle size of cassava flour. The average particle size of the particles decreased from 19.23 μm to 16.71 μm by 13.1%. A number of studies have shown that fermentation reduces the particle size of cassava flour (Isa et al. 2021; Kurniadi et al. 2019; Pi et al. 2021). The reason for this phenomenon may be due to the decomposition of the starch content in cassava by yeast fermentation, leading to a decrease in the particle size of fermented cassava flour (Zhao et al. 2019).

**TABLE 3:** Particle size distribution of cassava flour.

Particle size distribution	CK	Fermentation
D_10_ (μm)	7.27 ± 0.12	5.81 ± 0.09*
D_50_ (μm)	15.30 ± 0.05	13.10 ± 0.14*
D_90_ (μm)	44.32 ± 1.03	37.26 ± 0.66*
Average particle size (μm)	19.23 ± 0.27	16.71 ± 0.09*

*Note:* Data are expressed as mean ± standard deviation (SD) (*n* = 3). CK is for unfermented cassava flour, Fermentation is for fermented cassava flour.

* Indicates significant differences at *p <* 0.05.

#### 3.3.2 Viscosity Characteristics

**FIGURE 4:**
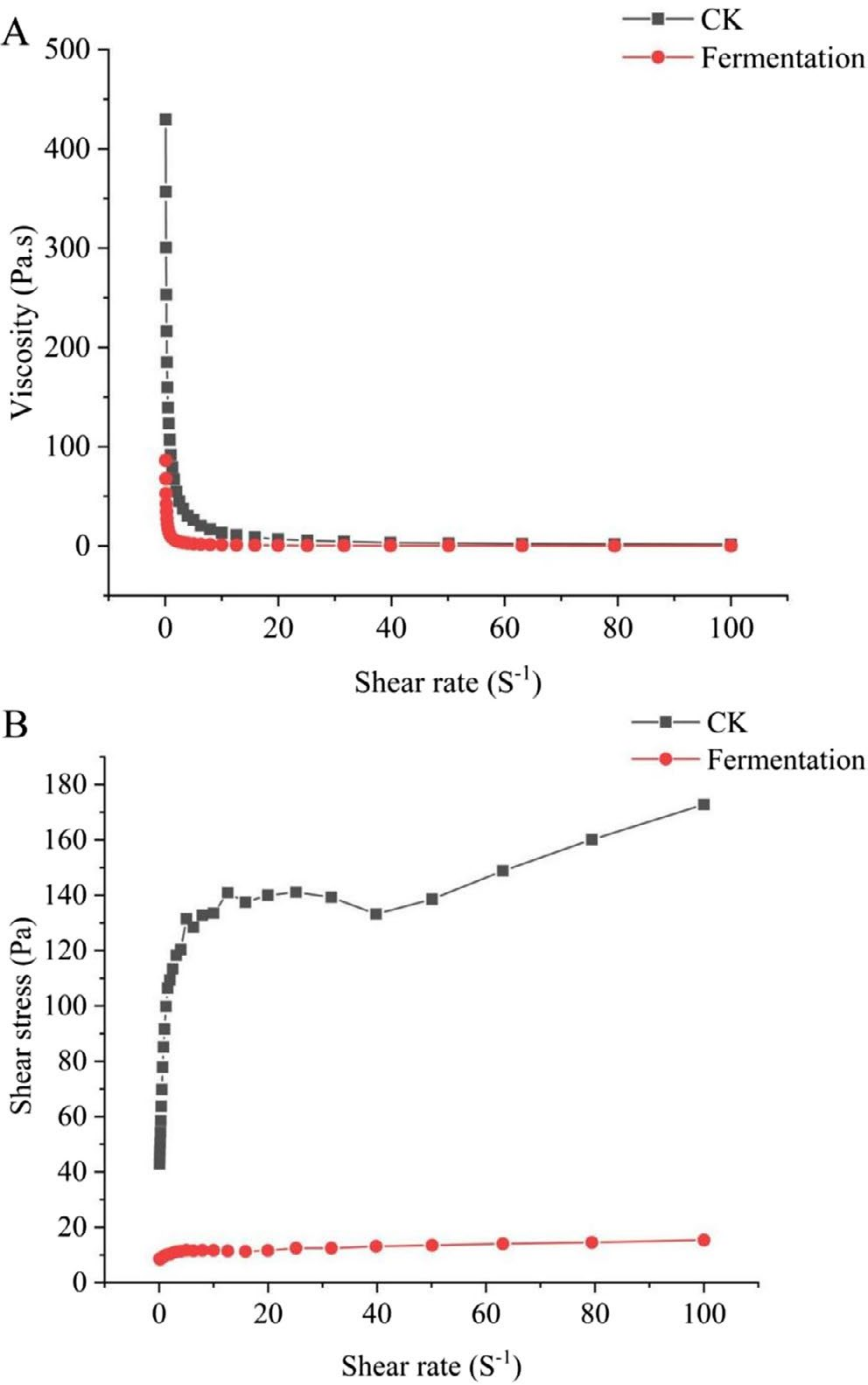
Characterization of the viscosity of cassava flour. (A) Viscosity of cassava flour, (B) Shear stress of cassava flour.

The viscosity of CK and fermented cassava flours decreased with increasing shear rate. At a low shear rate, the shear thinning phenomenon rapidly reduced the viscosity of both flours. With further increase in the shear rate, the viscosity decreased slowly and leveled off (Figure 4A). The shear stress required during the flow of both types of cassava flour increased with the increase of shear frequency, which is characteristic of pseudoplastic fluids (Bautista and Pérez 2010). The shear stress of fermented cassava flour paste increased at a slower rate than that of CK, which also indicated that the fermented cassava flour paste required less force under the same flow pattern, which reduced the difficulty of transporting and mixing the materials during food processing (Figure 4B). The reason for this phenomenon may be the cassava flour paste within the formation of wrapped water molecules of the three‐dimensional mesh structure; this mesh structure is formed by the starch and other polymer chain substances interlinked with each other entangled, increasing the intermolecular forces. Fermentation reduces the viscosity of cassava flour, probably because microbial fermentation changes the structure of cassava flour starch, weakening the network structure of cassava flour paste (Oh et al. 1999).

**FIGURE 5:**
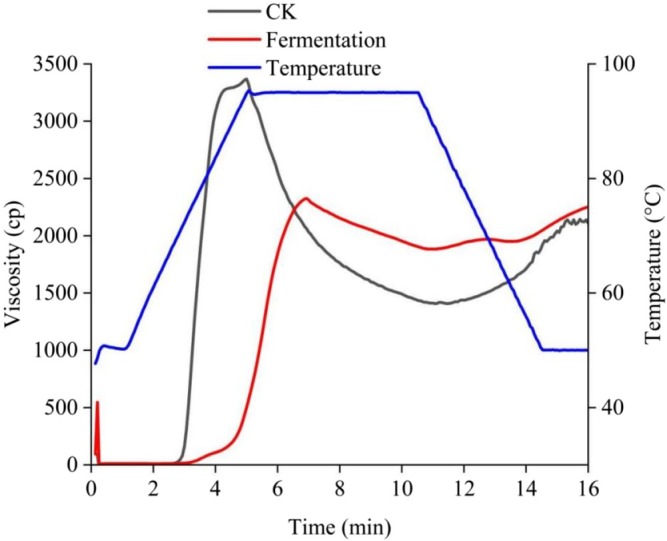
Diagram of viscometer characterization of cassava flour.

The peak viscosity (PV) and decay values of cassava flour changed after fermentation (Table 4; Figure 5). The final viscosity (FV) indicates the viscosity of cassava flour after cooling, and the decay value mainly reflects the thermal stability of cassava flour; the lower the decay value, the better the stability. The FV of fermented cassava flour was greater than that of CK (2105 cp), and the attenuation value of fermented cassava flour was smaller than that of CK, which indicates that fermentation improved the stability of cassava flour. Due to the breakage and debranching of amylopectin in the amorphous zone of starch after fermentation, the magnitude of the interaction force between the amorphous and crystalline zones of starch is altered to make it more soluble in hot water (Kresnowati et al. 2019; Niba et al. 2002). It is also possible that fermentation increases the protein content of cassava flour, which hinders swelling/starch‐water interactions, so that the PV value is reached later than the CK value; and that the destruction of starch and non‐starch components by fermentation (and therefore changes in granule size) affects the microstructure of the starch, which in turn reduces viscosity (Ali et al. 2024; Wu et al. 2023).

**TABLE 4:** Viscometer test parameters for cassava flour.

	Paste temperature (°C)	PV (cp)	Attenuation value	Valley viscosity (cp)	Regeneration value	FV (cp)
CK	95.35	3321	1884	1437	668	2105
Fermentation	95.00	2326	442	1884	427	2311

#### 3.3.3 Crystallinity

**FIGURE 6:**
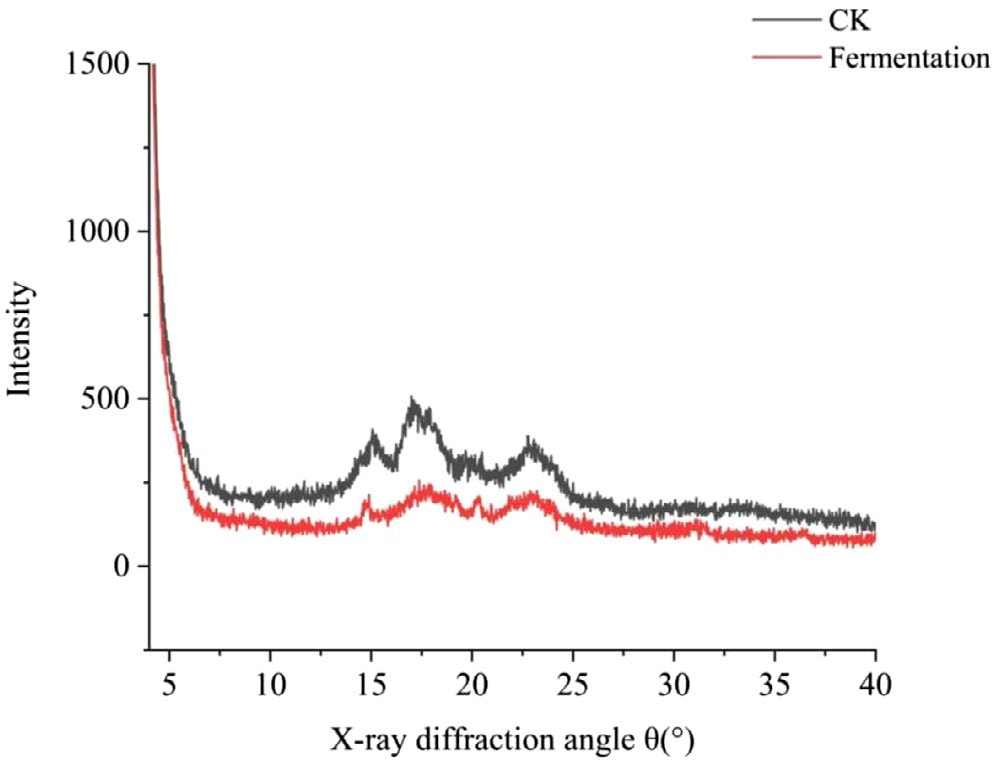
X‐ray diffraction patterns of cassava flour.

Traditional cassava flour is a C‐type starch, generally with characteristic peaks at diffraction angles 2θ of 15.1°, 17.0°, 17.9°, 20.1°, and 23.0°, and fermented cassava flour has characteristic peaks at diffraction angles 2θ of 14.9°, 17.9°, 20.2°, and 23.1°, whose crystalline forms are all C‐type, still maintaining the crystalline structure of the original cassava flour (Figure 6). Fermentation changed the diffraction intensity, but not the crystal type of cassava flour. The relative crystallinity decreased from 15.83% to 10.06% after fermentation. This phenomenon may be due to the action of enzymes or the hydrolysis of starch granules in the crystallization zone into small molecules by acids and enzyme substances produced by microbial fermentation, resulting in a decrease in crystallinity after fermentation (Gomes et al. 2004). In addition, a related study found that the fermented cassava flour diffractograms showed narrow peaks, which indicate the hydrolysis of the amorphous region during fermentation, thus leaving the crystalline region intact.

### 3.4 Characteristics of Cassava Bread

#### 3.4.1 Bread Awakening Height and Specific Volume

**FIGURE 7:**
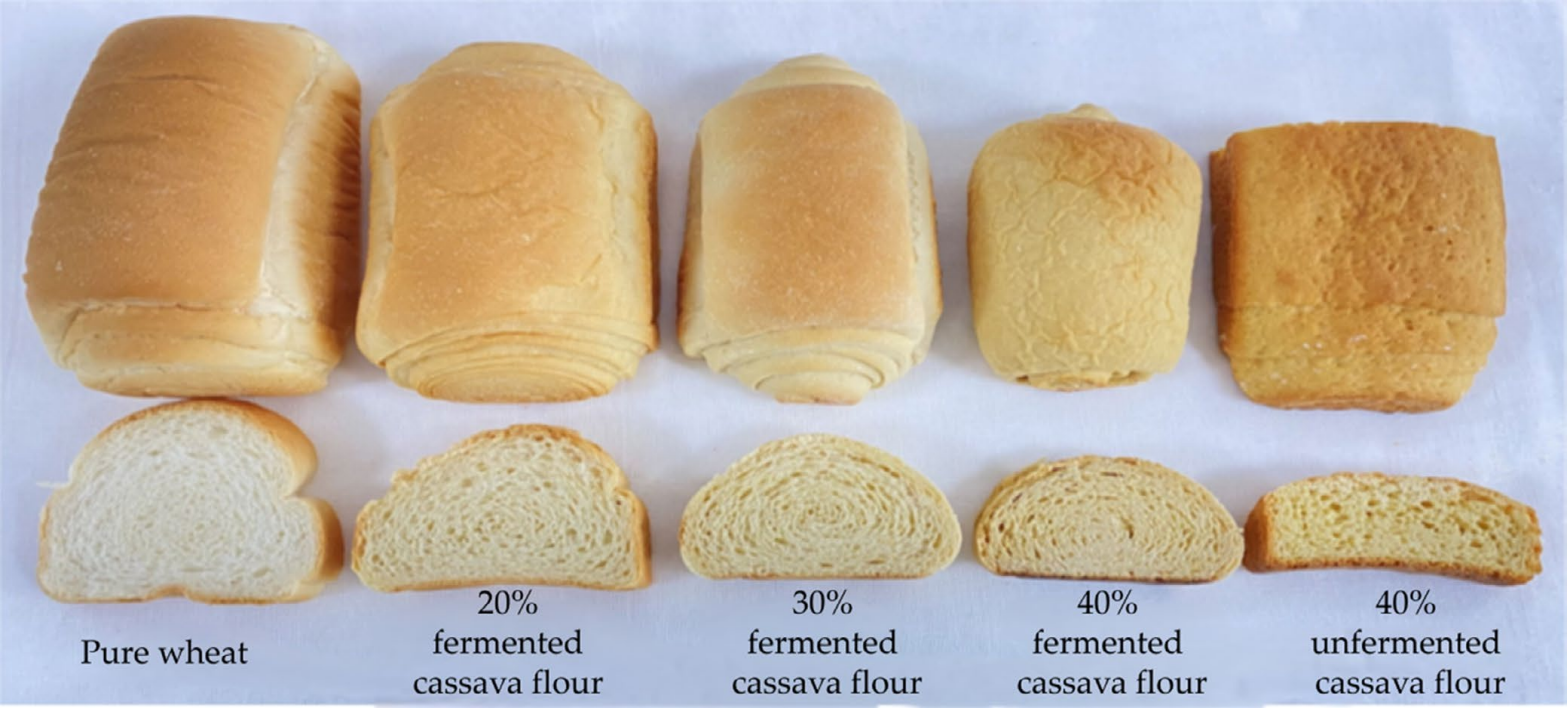
Awakening height and specific volume of cassava bread.

Awakening height and specific volume are important indicators of bread sensory, and the addition of cassava flour affects the change of these two indicators. With the increase of the amount of cassava flour added, the two indicators decreased significantly (*p* < 0.05), in which the bread with 40% fermented cassava flour added was significantly higher than that of the bread with added cassava flour (*p* < 0.05). The bread with 20% fermented cassava flour has a similar awakening height and specific volume to the pure wheat bread (Table 5; Figure 7). The addition of fermented cassava flour may cause the gluten protein in the dough to be diluted, or the enzymes produced by microbial fermentation or the increase in the acidity of the fermentation system may cause the starch to be degraded into soluble sugar, which is more absorbent, leading to a decrease in the water content bound to the gluten protein, and the dough's malleability may deteriorate, leading to a reduction in the amount of carbon dioxide retained during the fermentation process, resulting in the decrease of the internal structure of the bread's density and specific volume (Mudgil et al. 2016; Schopf and Scherf 2021).

**TABLE 5:** Awakening height and specific volume of cassava bread.

Breads	Awakening height (cm)	Mass (g)	Volumes (mL)	Specific volume (mL/g)
Pure wheat (CK)	8.2 ± 0.0^a^	147.1 ± 1.1^c^	825.0 ± 7.6^a^	5.6 ± 0.1^a^
20% fermented flour	7.3 ± 0.0^b^	156.8 ± 2.1^b^	608.3 ± 10.1^b^	3.9 ± 0.2^b^
30% fermented flour	6.2 ± 0.1^c^	158.8 ± 0.6^a^	500.3 ± 5.8^c^	3.2 ± 0.4^c^
40% fermented flour	4.8 ± 0.1^d^	161.1 ± 4.7^a^	395.7 ± 6.0^d^	2.5 ± 0.1^d^
40% flour	3.8 ± 0.0^e^	167.2 ± 1.8^b^	345.2 ± 2.8^e^	2.0 ± 0.2^e^

*Note:* Different lowercase letters in the same column indicate significant differences (*p <* 0.05).

#### 3.4.2 Bread Texture Characteristics

Bread texture characteristics include firmness, masticatory, and elasticity, which are important bread sensory indicators. The addition of cassava flour affects the change of these three indicators; with the increase of cassava flour, firmness and masticatory increased significantly, and elasticity decreased significantly (*p* < 0.05), whereas the three indicators of bread with 40% fermented cassava flour were significantly better than those with 40% fermented cassava flour (Table 6). This phenomenon may be because the particle size and viscosity of fermented cassava flour are less than CK, affecting the bread texture characteristics. In contrast, the viscosity has the most significant effect on the formation of the dough. It is crucial to maintain the appropriate viscosity for food processing because viscosity that is too large or too small is not conducive to the mixing and shaping of food in the processing process, so the addition of a large amount of fermented cassava flour will also reduce the quality of bread (Guo et al. 2022).

**TABLE 6:** Bread texture characteristics.

Breads	Firmness (*N*)	Masticatory (mj)	Elasticity (mm)
Pure wheat (CK)	4.40 ± 0.33^d^	18.43 ± 1.56^d^	10.14 ± 0.40^a^
20% fermented flour	5.27 ± 0.50^d^	20.43 ± 1.01^cd^	9.48 ± 0.17^a^
30% fermented flour	7.80 ± 0.59^c^	23.20 ± 2.20^c^	8.64 ± 0.44^b^
40% fermented flour	15.01 ± 0.59^b^	27.37 ± 1.31^b^	7.06 ± 0.39^c^
40% flour	18.14 ± 0.64^a^	37.17 ± 1.63^a^	5.58 ± 0.40^d^

*Note:* Different lowercase letters in the same column indicate significant differences (*p <* 0.05).

#### 3.4.3 Bread Sensory Evaluation

As the amount of fermented cassava flour increased, the shape of the bread gradually became less, and the shape score decreased. From the bread profile, the color gradually turned yellow, and the texture was firm, so the scores of color and internal organization gradually became lower, decreasing the overall sensory score. When the proportion of CK and fermented cassava flour was 40%, the sensory score of fermented cassava bread was higher than that of cassava bread because its shape and texture were better than those of CK, and it had a unique fermented flavor; this result was also consistent with the results of the texture meter (Table 7). In conclusion, bread with 20% fermented cassava flour has good quality and flavor, which is closest to pure wheat bread.

**TABLE 7:** Bread sensory evaluation.

Breads	Sensory scores
Pure wheat (CK)	91.4
20% fermented flour	83.6
30% fermented flour	69.6
40% fermented flour	48.7
40% flour (CK)	27.3

## 4 Conclusions

This study demonstrates that yeast fermentation significantly enhances the nutritional, functional, and sensory properties of cassava flour. The process increased the true protein content to 3.86 ± 0.12 g/100 g while reducing hydrocyanic acid to 7.40 ± 0.22 mg/kg, improving both nutritional value and safety. Fermentation generated key flavor compounds, including 2‐methyl‐1‐butanol, phenethyl alcohol, and ethyl linoleate, which contributed to a more desirable aroma profile. The modified cassava flour exhibited reduced particle size, viscosity, and crystallinity, leading to improved stability and processing performance. When incorporated into bread formulations, 20% fermented cassava flour produced products with texture, volume, and sensory qualities closely resembling wheat bread. These findings provide critical insights for developing high‐quality cassava‐based food products and support the industrial application of fermentation technology to enhance cassava utilization.

## Author Contributions

**Peixu Du:** conceptualization (equal), data curation (equal), formal analysis (equal), investigation (equal), software (equal), validation (equal), writing – original draft (equal), writing – review and editing (equal). **Jinxiao Liang:** data curation (equal), formal analysis (equal), investigation (equal), writing – review and editing (equal). **Hao Zhang:** data curation (equal), investigation (equal), software (equal), validation (equal). **Wanbi Tan:** data curation (equal), investigation (equal), validation (equal). **Guotao Liang:** supervision (equal). **Onguala Paul:** supervision (equal). **Quanfa Zhou:** supervision (equal). **Liming Lin:** supervision (equal). **Xingxing Dong:** conceptualization (equal), writing – review and editing (equal). **Zhenwen Zhang:** funding acquisition (equal), project administration (equal), resources (equal), supervision (equal), writing – review and editing (equal). **Yi He:** conceptualization (equal), data curation (equal), funding acquisition (equal), investigation (equal), methodology (equal), project administration (equal), resources (equal), supervision (equal), validation (equal), visualization (equal), writing – original draft (equal), writing – review and editing (equal).

## Conflicts of Interest

The authors declare no conflicts of interest.

## Supporting information


**Table S1.** Response substances and performance of each sensor of the electronic nose.

## Data Availability

The data that support the findings of this study are available on request from the corresponding author.
