# Supplementary material for: Enhanced Cassava Flour Quality to Improve the Cassava Bread by Attibutes Yeast Fermentation
Source: Food Sci Nutr. 2025 Jul 14;13(7):e70581. doi: 10.1002/fsn3.70581 (PMC12257496; doi:10.1002/fsn3.70581)
Supplement: Supplementary file 1 — Table S1. Response substances and performance of each sensor of the electronic nose. [file FSN3-13-e70581-s001.docx]

Table S1. Response substances and performance of each sensor of the electronic nose.

| Sensors | Response substance | Performance description |
| --- | --- | --- |
| S1 | Short-chain alkanes | Propane, smoke, etc. |
| S2 | Carbonaceous substances, etc. | Alcohol, fumes, isobutane, formaldehyde, etc. |
| S3 | Hydrogen | Hydrogen, hydrogen-containing gases, etc. |
| S4 | Sulfides | Hydrogen sulfide, sulfur dioxide, etc. |
| S5 | Nitrogenous substances | Ammonia, amines, etc. |
| S6 | Aldehydes and ketones | Toluene, acetone, ethanol hydrogen, etc. |
| S7 | Flammable gases from short-chain alkanes | Methane, natural gas, biogas, etc. |
| S8 | Liquefied gas | Liquefied gas |
| S9 | Alkanes, alcohols, ketones, etc. | Toluene, formaldehyde, benzene, alcohol, acetone, etc. |
| S10 | Hydrogen and hydrogen-containing substances | Hydrogen |
| S11 | Alkanes, carbon monoxide, etc. | Liquefied petroleum gas, alkanes, etc. |
| S12 | Some organic solvents | Liquefied petroleum gas, methane, etc. |
| S13 | Short-chain alkanes | Methane |
| S14 | Short-chain alkanes | Methane, gas, smoke, etc. |
| S15 | Nitrogenous substances | Ammonia, amines, etc. |
| S16 | Sulfur sensitive to chemicals | Hydrogen sulfide, sulfide, etc. |
| S17 | Hydrogen-containing substances | Hydrogen-containing substances, such as hydrogen |
| S18 | Alcohol, some organic solvents, etc. | Aromatic hydrocarbons, aliphatic hydrocarbons, alicyclic hydrocarbons, halogenated hydrocarbons, ethers, esters, diol derivatives, acetonitrile, pyridine, phenol, etc. |
|  |  |  |
| S19 | Alcohols, aldehydes, ketones and benzene | Alcohols, ketones, aldehydes, aromatic compounds, etc. |
| S20 | Short-chain alkanes | Methane, biogas, natural gas, etc. |
| S21 | Combustible gases, etc. | Combustible gases, etc. |
| S22 | Volatile organic compounds | Phenols, ketones, ethyl acetate, cyclohexanone, chlorobenzene, methylbenzene, ethers, etc. |
| S23 | Alkanes, olefins, aromatics, etc. | LPG, natural gas, gas, propane, propylene, butane, butylene, etc. |
| S24 | Alkanes, olefins, hydrogen, etc. | Liquefied gases, combustible gases, etc. |
| S25 | Alkanes, carbon monoxide, alkenes, alcohols, nitrogen oxides, ketones, aldehydes, etc. | Alkanes, alcohols, natural gas, fumes, etc. |
|  |  |  |
| S26 | Some organic solvents | Alcohols, ethers, esters, ketones, aromatic hydrocarbons, aliphatic hydrocarbons, alicyclic hydrocarbons, and halogenated hydrocarbons |
|  |  |  |
| S27 | Sulfides, nitrides, carbides, hydrocarbons, nitrogen oxides, etc. | Smoke, cooking odors, etc. |
|  |  |  |
| S28 | Short-chain alkanes | Methane, gas, etc. |
